# Hepatic lipid quantity and quality in type 2 diabetes mellitus

**DOI:** 10.3389/fphar.2026.1816958

**Published:** 2026-05-29

**Authors:** Kahori Shimizu, Hideo Shindou, Koji Tomita, Hiroyuki Mizuguchi

**Affiliations:** 1 Laboratory of Biochemistry and Molecular Biology, Graduate School of Pharmaceutical Sciences, The University of Osaka, Osaka, Japan; 2 Department of Lipid Life Science, National Institute of Global Health and Medicine, Japan Institute for Health Security, Tokyo, Japan; 3 Department of Medical Lipid Science, Graduate School of Medicine, The University of Tokyo, Tokyo, Japan; 4 Laboratory of Molecular Biology, Faculty of Pharmacy, Osaka Ohtani University, Osaka, Japan; 5 Global Center for Medical Engineering and Informatics, The University of Osaka, Osaka, Japan; 6 Integrated Frontier Research for Medical Science Division, Institute for Open and Transdisciplinary Research Initiatives, The University of Osaka, Osaka, Japan; 7 Center for Infectious Disease Education and Research, The University of Osaka, Osaka, Japan

**Keywords:** lipid intermediate, lipid quality, lipid quantity, phospholipid diversity, type 2 diabetes mellitus

## Abstract

The incidence of type 2 diabetes mellitus (T2DM) has been increasing worldwide. In the pathophysiology of metabolic diseases, including T2DM, both the quantity and quality of lipids that accumulate in the body have attracted significant research attention. Excess lipids accumulate in non-adipose tissues, such as the liver and skeletal muscles, leading to insulin resistance and an increased risk of developing T2DM. The liver is the central organ for glucose and lipid metabolism, and impaired hepatic regulatory mechanisms lead to hyperglycemia, insulin resistance, and hepatic lipid accumulation. Hepatic triglyceride (TG) accumulation contributes to the development of hepatic insulin resistance; however, the quantity of TGs alone is insufficient to explain the development of glucose and lipid metabolic dysfunctions. Thus, we focus on lipid quality, particularly fatty acids. In addition to TGs, phospholipids store fatty acids, which vary in carbon chain length, number of double bonds, and position of double bonds (such as ω3 and ω6), resulting in lipid diversity. Changes in the fatty acid chains of phospholipids are also strongly associated with glucose and lipid metabolism. In this mini review, we discuss the involvement of hepatic lipid quantity and quality in T2DM, with the aim of contributing to the development of novel T2DM therapies.

## 1 Introduction

The global incidence of diabetes mellitus, a chronic heterogeneous metabolic disease, has increased in recent years. According to the International Diabetes Federation Diabetes Atlas, 11th Edition, the global prevalence of diabetes in individuals aged 20–79 years was 588.7 million individuals in 2024, exceeding the combined population of the U.S., Canada, Mexico, and the Caribbean. Type 2 diabetes mellitus (T2DM), which accounts for over 90% of all diabetes cases, is characterized by insulin resistance and β-cell dysfunction (Muoio and Newgard, 2008; Banday et al., 2020). In the pathophysiology of metabolic diseases, including T2DM, both the quantity and quality of lipids (hereafter referred to as lipid quantity and quality) accumulated in the body have attracted attention. Excess lipids accumulate in non-adipose tissues such as the liver and skeletal muscle (Britton and Fox, 2011; Suganami et al., 2012). The liver is a principal metabolic organ that plays a crucial role in regulating glucose and lipid homeostasis. Hepatic lipid accumulation, a hallmark of metabolic dysfunction-associated steatotic liver disease (MASLD), is strongly associated with the onset of insulin resistance (Samuel and Shulman, 2016) and an increased risk of T2DM (Mantovani et al., 2021).

Triglycerides (TGs) are the predominant lipid class stored in lipid droplets, and the quantity of lipids is a component of the MASLD grading system (Marra and Svegliati-Baroni, 2018). Hepatic TG accumulation contributes to the development of hepatic insulin resistance (Samuel and Shulman, 2016). However, the total amount of TGs stored in the liver is not the major determinant of lipotoxicity, as TGs are relatively inert storage forms. Rather, lipid intermediates are thought to play a key role. For example, diacylglycerols (DAGs) can activate protein kinase Cε and impair insulin receptor signaling. Lipotoxicity refers to the toxic effects of excessive lipid accumulation in non-adipose tissues, leading to cellular dysfunction and metabolic disturbances. Consistent with this, lipid intermediates, such as DAGs, lysophosphatidylcholine, and ceramides, exhibit toxicity and interfere with insulin signaling (Marra and Svegliati-Baroni, 2018). In addition, glycerophospholipids (phospholipids) in biological membranes have recently attracted attention as dynamic regulators of cellular metabolism and signaling (Hishikawa et al., 2014; Lamari et al., 2025). Phospholipids are biosynthesized from DAG and lysophospholipids through the Kennedy pathway and Lands cycle, respectively. Phospholipids consist of two fatty acids and one polar head group linked to a glycerol backbone. Changes in the fatty acid chains of phospholipids have been implicated in glucose and lipid metabolism (Valentine et al., 2022). In this mini review, we focus on the emerging concept that both lipid quantity and quality in the liver are associated with glucose and lipid metabolism in T2DM.

## 2 Hepatic lipid quantity and metabolic dysfunction

Hepatic TG accumulation is strongly associated with the induction of insulin resistance and impaired glucose metabolism. Both experimental and clinical studies have demonstrated a close correlation between intrahepatic lipid content and metabolic abnormalities, including elevated fasting glucose levels, increased hepatic glucose production, and reduced insulin sensitivity (Samuel et al., 2004; Kotronen et al., 2007; Fabbrini et al., 2009; Fabbrini et al., 2010; Lim et al., 2011). Elevated hepatic lipid content is posited to impair glucose metabolism by disrupting insulin signaling pathways and enhancing hepatic glucose production. The prevalence of MASLD, formerly known as nonalcoholic fatty liver disease, in patients with T2DM is estimated to be approximately 60%–70% (Younossi et al., 2024). These observations have established that hepatic lipid accumulation is closely linked to T2DM.

Experimental evidence further supports a causal relationship between hepatic TG content and glucose metabolism. Pharmacological interventions have shown that reducing hepatic TG content frequently leads to improvements in glucose metabolism. For example, bezafibrate treatment reduced hepatic steatosis and improved hepatic insulin sensitivity in diabetic TallyHo mice (Franko et al., 2017). Empagliflozin, a specific sodium–glucose cotransporter 2 inhibitor, protects mice against diet-induced obesity, hepatic steatosis, and insulin resistance (Radlinger et al., 2023). In patients with MASH, pioglitazone administration significantly decreased hepatic lipid content and increased hepatic insulin sensitivity (Belfort et al., 2006). In genetic interventions, liver-specific overexpression of lipoprotein lipase, which plays a major role in lipid metabolism through the hydrolysis of TG in chylomicrons and very low-density lipoproteins, improved glucose metabolism in high-fat diet-fed mice (Shimizu et al., 2022). In this model, hepatic overexpression of lipoprotein lipase decreased lipid droplet formation in the liver and improved fasting blood glucose levels. These findings indicate that hepatic lipid quantity influences glucose metabolism and contributes to T2DM. Although TGs constitute the predominant lipid class within droplets and the abundance of lipids is integral to the grading system of MASLD, lipids, as signaling intermediates, encompass a diverse array of molecules with distinct functions. Lipid intermediates and lipid composition, such as fatty acids, may impair glucose metabolism. These observations highlight the importance of lipid quantity and quality in regulating glucose metabolism.

## 3 Hepatic lipid quality and metabolic regulation

### 3.1 Lipid intermediates

Although hepatic TG accumulation is strongly associated with insulin resistance, increasing evidence indicates that TG itself is not the primary lipid responsible for metabolic dysfunction. Rather, lipid storage in the form of TG is considered protective against lipotoxicity (Marra and Svegliati-Baroni, 2018; Geng et al., 2021). TG synthesis facilitates the sequestration of excess fatty acids into lipid droplets, thereby limiting the accumulation of potentially toxic lipid intermediates such as DAGs and ceramides. Consistent with this concept, inhibition of TG synthesis has been shown to exacerbate oxidative stress, inflammation, and fibrosis (Yamaguchi et al., 2007).

DAG, a lipid intermediate comprising glycerol and two fatty acids, has been identified as a key mediator of hepatic insulin resistance. Although intrahepatic DAG levels are increased in MASLD (Geng et al., 2021), their metabolic impact depends primarily on subcellular localization rather than total cellular abundance. In particular, DAG accumulation within membrane compartments represents the bioactive pool that promotes the recruitment, activation, and translocation of protein kinase Cε (PKCε), the predominant PKC isoform in the liver, to the plasma membrane, thereby impairing insulin signaling (Perry et al., 2014; Samuel and Shulman, 2016; Samuel and Shulman, 2018). PKCε activation reduces insulin-stimulated phosphorylation of insulin receptor substrate-2 (IRS2), IRS2-associated phosphatidylinositol 3-kinase activity, and phosphorylation of Akt2, leading to impaired suppression of gluconeogenesis (Perry et al., 2014). Moreover, insulin resistance further exacerbates DAG accumulation, creating a vicious metabolic cycle. Impaired insulin signaling enhances adipose tissue lipolysis, increasing the influx of fatty acids to the liver, and promotes *de novo* lipogenesis, both of which contribute to increased hepatic DAG levels and further activation of PKCε (Perry et al., 2014; Samuel and Shulman, 2016; Samuel and Shulman, 2018). Collectively, these findings indicate that hepatic DAG accumulation is a critical mediator in the pathogenesis of hepatic insulin resistance, a key component of metabolic dysfunction.

Ceramides, precursors of sphingomyelin, represent another class of bioactive lipids implicated in insulin resistance, inflammation, oxidative stress, and cell death (Marra and Svegliati-Baroni, 2018). Ceramide levels are increased in MASLD and T2DM due to enhanced *de novo* ceramide synthesis driven by excess fatty acid availability, particularly saturated fatty acids, as well as alterations in sphingolipid metabolism (Chavez and Summers, 2012). Accumulation of ceramides activates various signaling pathways, leading to disruption of normal cellular functions, including insulin activity. Ceramides directly inhibit Akt phosphorylation, induce endoplasmic reticulum stress, and cause mitochondrial dysfunction (Chavez and Summers, 2012; Chaurasia and Summers, 2015). In particular, a specific ceramide species, C16:0-ceramide, has been implicated as a key mediator of insulin resistance pathophysiology (Raichur et al., 2014; Turpin et al., 2014). Pharmacological and genetic interventions have shown that the inhibition of *de novo* ceramide synthesis, for example, using serine palmitoyltransferase inhibitors such as myriocin, significantly improves several metabolic parameters, including insulin resistance, atherosclerosis, and cardiomyopathy (Holland and Summers, 2008). These findings highlight the importance of ceramide metabolism in the regulation of hepatic insulin sensitivity.

In addition to DAGs and ceramides, other lipid species, such as lysophosphatidylcholine and specific fatty acid species, have been implicated in the regulation of metabolic processes. Alterations in the composition and distribution of these lipid species may disrupt cellular homeostasis and contribute to metabolic abnormalities. Moreover, the quality of fatty acids plays an important role in determining metabolic outcomes. Saturated fatty acids, such as palmitate, promote ceramide synthesis and are more likely to induce lipotoxicity and insulin resistance, whereas monounsaturated fatty acids, such as oleate, favor TG storage and may reduce the accumulation of lipotoxic intermediates, including DAGs and ceramides (Listenberger et al., 2003; Chavez and Summers, 2012). Collectively, these observations indicate that the metabolic consequences of hepatic lipid accumulation depend not only on the total quantity of stored lipids but also on their molecular composition, intracellular distribution, and partitioning into distinct lipid species.

### 3.2 Phospholipid diversity

Phospholipids, along with cholesterol, are major lipid components of cellular membranes. Their basic structure consists of a glycerol backbone, a polar headgroup, and two hydrophobic fatty acyl chains, which support lipid bilayer formation and cellular functions. Phospholipid diversity is primarily attributed to combinatorial variations in polar headgroups and fatty acids. Phosphatidic acid contains a phosphate group as its polar headgroup. Other phospholipid classes are formed through esterification of the phosphate group with molecules such as choline, ethanolamine, serine, inositol, or glycerol, giving rise to the major phospholipid classes phosphatidylcholine (PC), phosphatidylethanolamine, phosphatidylserine, phosphatidylinositol, and phosphatidylglycerol, respectively, as well as cardiolipin, which has a unique diphosphatidylglycerol-based structure (Valentine et al., 2023). The fatty acid chains of phospholipids are typically 12–24 carbons in length and contain 0–6 double bonds. These fatty acids differ in carbon chain length, double-bond numbers, and double-bond positions, such as ω3 and ω6. As a result, phospholipids comprise >1,000 molecular species arising from variations in polar headgroups and fatty acyl chains. Phospholipid composition contributes to membrane properties (van Meer et al., 2008; Harayama and Riezman, 2018).

**FIGURE 1:**
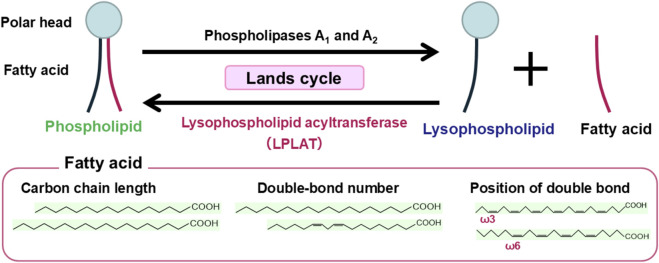
Lands cycle and phospholipid diversity. Phospholipids are cleaved by phospholipases A_1_ and A_2_ to form lysophospholipids, which are subsequently remodeled back to phospholipids by lysophospholipid acyltransferases. The fatty acids in phospholipids differ in carbon number, number of double bonds, and types of double bonds, thus creating phospholipid diversity.

Phospholipids undergo fatty acyl chain remodeling through the Lands cycle (Lands, 1958). In this cycle, phospholipases A_1_ and A_2_ cleave fatty acids from phospholipids, generating lysophospholipids. These lysophospholipids are subsequently reacylated to form phospholipids by lysophospholipid acyltransferases (LPLATs) (Figure 1) (Shindou and Shimizu, 2009; Kita et al., 2019; Valentine et al., 2022). To date, 14 mammalian LPLATs have been identified. LPLATs contribute to phospholipid diversity and are involved in glucose and lipid metabolism.

LPLAT12, also known as lysophosphatidylcholine acyltransferase 3 (LPCAT3) or membrane-bound *O*-acyltransferase domain-containing 5 (MBOAT5), is a major hepatic LPLAT that preferentially incorporates arachidonic acid into PC to generate polyunsaturated PCs (Hishikawa et al., 2008; Hashidate-Yoshida et al., 2015; Rong et al., 2015). LPLAT12 deficiency reduces arachidonic acid-containing PC, resulting in impaired very low-density lipoprotein secretion (Hashidate-Yoshida et al., 2015; Tian et al., 2023b). Liver-specific knockout of LPLAT12 improves insulin sensitivity (Tian et al., 2023a; Tian et al., 2023b). In addition, the administration of antisense oligonucleotides against LPLAT12 improves obesity and insulin sensitivity in both high-fat diet–fed and ob/ob mice (Tian et al., 2023b). These findings indicate that LPLAT12-mediated membrane phospholipid composition is a novel regulator of insulin sensitivity. Although alterations in LPLAT12 expression or activity in T2DM are not fully established, these observations suggest that reduced incorporation of arachidonic acid into membrane phospholipids may alter membrane properties and lipid signaling, thereby influencing insulin sensitivity.

LPLAT11, also known as lysophosphatidylinositol acyltransferase 1 or MBOAT7 (Lee et al., 2008; Lee et al., 2012), is a significant genetic modifier in fatty liver diseases. The rs641738C > T genetic variant, located near LPLAT11, is associated with reduced hepatic LPLAT11 expression. In the context of fatty liver disease, this variant is linked to an increased risk of MASLD, including hepatic lipid accumulation and fibrosis (Mancina et al., 2016; Teo et al., 2021). Hepatocyte-specific LPLAT11-knockout mice exhibited spontaneous development of steatosis and hepatic fibrosis when fed a high-fat diet (Tanaka et al., 2021). Depletion of LPLAT11 in hepatocytes increases phosphatidylinositol turnover, leading to sustained generation of DAG, a key intermediate for TG synthesis (Tanaka et al., 2021). While increased DAG generation in LPLAT11-deficient hepatocytes may contribute to TG synthesis and hepatic lipid accumulation, its direct impact on insulin sensitivity remains to be fully elucidated. These findings provide insights into the pathogenesis of MASLD and may inform the development of therapeutic strategies.

Recent experimental evidence has revealed that modulation of hepatic LPLAT improves glucose metabolism. Overexpression of LPLAT10, also known as LPCAT4 and lysophosphatidylethanolamine acyltransferase 2 (Cao et al., 2008; Eto et al., 2020), in the liver has been shown to attenuate postprandial hyperglycemia by enhancing glucose-stimulated insulin secretion in mice (Shimizu et al., 2024). In this model, liver-specific LPLAT10 overexpression altered hepatic and serum phospholipid composition and improved glucose metabolism without affecting body weight or food intake. The underlying mechanism is thought to involve changes in hepatic phospholipid composition that alter circulating phospholipid species, which may act on pancreatic β-cells to enhance glucose-stimulated insulin secretion. These observations indicate that changes in phospholipid composition in the liver can influence systemic metabolic homeostasis, potentially through the modulation of membrane composition, intracellular signaling, and inter-organ metabolic communication. However, pharmacological agents that specifically target LPLATs remain limited. Although several experimental and genetic approaches have been used to modulate LPLAT activity in preclinical studies, their effects on insulin sensitivity are still under investigation, highlighting the need for the development of selective LPLAT modulators as potential therapeutic strategies for metabolic diseases.

**FIGURE 2:**
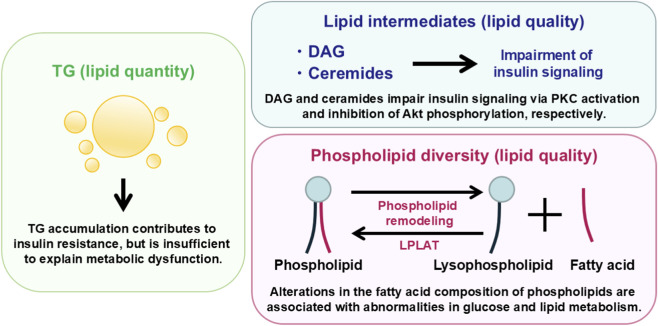
Lipid quantity and quality in metabolic regulation. TG accumulation represents lipid quantity, whereas lipid quality is defined by lipid intermediates and phospholipid composition, which are regulated by phospholipid remodeling pathways.

Collectively, these lipid-mediated mechanisms contribute to insulin resistance and insulin secretion in T2DM, although their relative impact may differ. Accumulation of DAG and subsequent activation of PKCε represent a central mechanism that directly impairs hepatic insulin signaling. Ceramides also play an important role by modulating cellular stress responses and inhibiting insulin signaling pathways. In parallel, alterations in phospholipid remodeling and fatty acid composition may exert more modulatory effects by influencing membrane properties, lipid signaling, and inter-organ communication. These pathways are interconnected and collectively contribute to the development and progression of metabolic dysfunction (Figure 2).

## 4 Conclusion

In this mini review, we focused on hepatic lipids as a key factor in understanding the pathology of T2DM and as a novel therapeutic target. Hepatic lipids are mainly composed of TGs; however, the quantity of TGs alone is insufficient to explain the development of glucose and lipid metabolic dysfunction. Lipid intermediates, such as DAGs and ceramides, also play important roles in abnormalities of glucose and lipid metabolism. Phospholipid diversity results from differences in their head groups and fatty acid chains. Alterations in the fatty acid composition of phospholipids are associated with abnormalities in glucose and lipid metabolism. In contrast, controlled modification of phospholipid fatty acid composition may improve metabolic regulation, thereby providing a potential therapeutic strategy for T2DM. Thus, both lipid quality and quantity should be considered in the development of novel treatments for T2DM.

## References

[B1] Banday M. Z. Sameer A. S. Nissar S. (2020). Pathophysiology of diabetes: an overview. Avicenna J. Med. 10 (4), 174–188. 10.4103/ajm.ajm_53_20 33437689 PMC7791288

[B2] Belfort R. Harrison S. A. Brown K. Darland C. Finch J. Hardies J. (2006). A placebo-controlled trial of pioglitazone in subjects with nonalcoholic steatohepatitis. N. Engl. J. Med. 355 (22), 2297–2307. 10.1056/NEJMoa060326 17135584

[B3] Britton K. A. Fox C. S. (2011). Ectopic fat depots and cardiovascular disease. Circulation 124 (24), e837–e841. 10.1161/CIRCULATIONAHA.111.077602 22156000

[B4] Cao J. Shan D. Revett T. Li D. Wu L. Liu W. (2008). Molecular identification of a novel Mammalian brain isoform of acyl-CoA:lysophospholipid acyltransferase with prominent ethanolamine lysophospholipid acylating activity, LPEAT2. J. of Biol. Chem. 283 (27), 19049–19057. 10.1074/jbc.M800364200 18458083

[B5] Chaurasia B. Summers S. A. (2015). Ceramides - lipotoxic inducers of metabolic disorders. Trends Endocrinol. Metab. 26 (10), 538–550. 10.1016/j.tem.2015.07.006 26412155

[B6] Chavez J. A. Summers S. A. (2012). A ceramide-centric view of insulin resistance. Cell Metab. 15 (5), 585–594. 10.1016/j.cmet.2012.04.002 22560211

[B7] Eto M. Shindou H. Yamamoto S. Tamura-Nakano M. Shimizu T. (2020). Lysophosphatidylethanolamine acyltransferase 2 (LPEAT2) incorporates DHA into phospholipids and has possible functions for fatty acid-induced cell death. Biochem. Biophys. Res. Commun. 526 (1), 246–252. 10.1016/j.bbrc.2020.03.074 32204912

[B8] Fabbrini E. Magkos F. Mohammed B. S. Pietka T. Abumrad N. A. Patterson B. W. (2009). Intrahepatic fat, not visceral fat, is linked with metabolic complications of obesity. Proc. of the Natl. Acad. of Sci. of the U. S. A. 106 (36), 15430–15435. 10.1073/pnas.0904944106 19706383 PMC2741268

[B9] Fabbrini E. Sullivan S. Klein S. (2010). Obesity and nonalcoholic fatty liver disease: biochemical, metabolic, and clinical implications. Hepatology 51 (2), 679–689. 10.1002/hep.23280 20041406 PMC3575093

[B10] Franko A. Neschen S. Rozman J. Rathkolb B. Aichler M. Feuchtinger A. (2017). Bezafibrate ameliorates diabetes via reduced steatosis and improved hepatic insulin sensitivity in diabetic TallyHo mice. Mol. Metab. 6 (3), 256–266. 10.1016/j.molmet.2016.12.007 28271032 PMC5323884

[B11] Geng Y. Faber K. N. de Meijer V. E. Blokzijl H. Moshage H. (2021). How does hepatic lipid accumulation lead to lipotoxicity in nonalcoholic fatty liver disease? Hepatol. Int. 15 (1), 21–35. 10.1007/s12072-020-10121-2 33548031 PMC7886759

[B12] Harayama T. Riezman H. (2018). Understanding the diversity of membrane lipid composition. Nat. Rev. Mol. Cell Biol. 19 (5), 281–296. 10.1038/nrm.2017.138 29410529

[B13] Hashidate-Yoshida T. Harayama T. Hishikawa D. Morimoto R. Hamano F. Tokuoka S. M. (2015). Fatty acid remodeling by LPCAT3 enriches arachidonate in phospholipid membranes and regulates triglyceride transport. Elife 4. 10.7554/eLife.06328 25898003 PMC4436788

[B14] Hishikawa D. Shindou H. Kobayashi S. Nakanishi H. Taguchi R. Shimizu T. (2008). Discovery of a lysophospholipid acyltransferase family essential for membrane asymmetry and diversity. Proc. Natl. Acad. Sci. U. S. A. 105 (8), 2830–2835. 10.1073/pnas.0712245105 18287005 PMC2268545

[B15] Hishikawa D. Hashidate T. Shimizu T. Shindou H. (2014). Diversity and function of membrane glycerophospholipids generated by the remodeling pathway in Mammalian cells. J. Lipid Res. 55 (5), 799–807. 10.1194/jlr.R046094 24646950 PMC3995458

[B16] Holland W. L. Summers S. A. (2008). Sphingolipids, insulin resistance, and metabolic disease: new insights from *in vivo* manipulation of sphingolipid metabolism. Endocr. Rev. 29 (4), 381–402. 10.1210/er.2007-0025 18451260 PMC2528849

[B17] Kita Y. Shindou H. Shimizu T. (2019). Cytosolic phospholipase A(2) and lysophospholipid acyltransferases. Biochim. Biophys. Acta Mol. Cell Biol. Lipids 1864 (6), 838–845. 10.1016/j.bbalip.2018.08.006 30905348

[B18] Kotronen A. Vehkavaara S. Seppala-Lindroos A. Bergholm R. Yki-Jarvinen H. (2007). Effect of liver fat on insulin clearance. Am. J. Physiol. Endocrinol. Metab. 293 (6), E1709–E1715. 10.1152/ajpendo.00444.2007 17895288

[B19] Lamari F. Rossignol F. Mitchell G. A. (2025). Glycerophospholipids: roles in cell trafficking and associated inborn errors. J. Inherit. Metab. Dis. 48 (2), e70019. 10.1002/jimd.70019 40101691 PMC11919462

[B20] Lands W. E. (1958). Metabolism of glycerolipides; a comparison of lecithin and triglyceride synthesis. J. Biol. Chem. 231 (2), 883–888. 10.1016/S0021-9258(18)70453-5 13539023

[B21] Lee H. C. Inoue T. Imae R. Kono N. Shirae S. Matsuda S. (2008). *Caenorhabditis elegans* mboa-7, a member of the MBOAT family, is required for selective incorporation of polyunsaturated fatty acids into phosphatidylinositol. Mol. Biol. Cell 19 (3), 1174–1184. 10.1091/mbc.e07-09-0893 18094042 PMC2262980

[B22] Lee H. C. Inoue T. Sasaki J. Kubo T. Matsuda S. Nakasaki Y. (2012). LPIAT1 regulates arachidonic acid content in phosphatidylinositol and is required for cortical lamination in mice. Mol. Biol. Cell 23 (24), 4689–4700. 10.1091/mbc.E12-09-0673 23097495 PMC3521678

[B23] Lim E. L. Hollingsworth K. G. Aribisala B. S. Chen M. J. Mathers J. C. Taylor R. (2011). Reversal of type 2 diabetes: normalisation of beta cell function in association with decreased pancreas and liver triacylglycerol. Diabetologia 54 (10), 2506–2514. 10.1007/s00125-011-2204-7 21656330 PMC3168743

[B24] Listenberger L. L. Han X. Lewis S. E. Cases S. Farese R. V. Jr. Ory D. S. (2003). Triglyceride accumulation protects against fatty acid-induced lipotoxicity. Proc. Natl. Acad. Sci. U. S. A. 100 (6), 3077–3082. 10.1073/pnas.0630588100 12629214 PMC152249

[B25] Mancina R. M. Dongiovanni P. Petta S. Pingitore P. Meroni M. Rametta R. (2016). The MBOAT7-TMC4 variant rs641738 increases risk of nonalcoholic Fatty liver disease in individuals of European descent. Gastroenterology 150 (5), 1219–1230 e1216. 10.1053/j.gastro.2016.01.032 26850495 PMC4844071

[B26] Mantovani A. Petracca G. Beatrice G. Tilg H. Byrne C. D. Targher G. (2021). Nonalcoholic fatty liver disease and risk of incident diabetes mellitus: an updated meta-analysis of 501 022 adult individuals. Gut 70 (5), 962–969. 10.1136/gutjnl-2020-322572 32938692

[B27] Marra F. Svegliati-Baroni G. (2018). Lipotoxicity and the gut-liver axis in NASH pathogenesis. J. Hepatol. 68 (2), 280–295. 10.1016/j.jhep.2017.11.014 29154964

[B28] Muoio D. M. Newgard C. B. (2008). Mechanisms of disease:molecular and metabolic mechanisms of insulin resistance and beta-cell failure in type 2 diabetes. Nat. Rev. Mol. Cell Biol. 9 (3), 193–205. 10.1038/nrm2327 18200017

[B29] Perry R. J. Samuel V. T. Petersen K. F. Shulman G. I. (2014). The role of hepatic lipids in hepatic insulin resistance and type 2 diabetes. Nature 510 (7503), 84–91. 10.1038/nature13478 24899308 PMC4489847

[B30] Radlinger B. Ress C. Folie S. Salzmann K. Lechuga A. Weiss B. (2023). Empagliflozin protects mice against diet-induced obesity, insulin resistance and hepatic steatosis. Diabetologia 66 (4), 754–767. 10.1007/s00125-022-05851-x 36525084 PMC9947060

[B31] Raichur S. Wang S. T. Chan P. W. Li Y. Ching J. Chaurasia B. (2014). CerS2 haploinsufficiency inhibits beta-oxidation and confers susceptibility to diet-induced steatohepatitis and insulin resistance. Cell Metab. 20 (5), 919. 10.1016/j.cmet.2014.10.007 29665397

[B32] Rong X. Wang B. Dunham M. M. Hedde P. N. Wong J. S. Gratton E. (2015). Lpcat3-dependent production of arachidonoyl phospholipids is a key determinant of triglyceride secretion. Elife 4. 10.7554/eLife.06557 25806685 PMC4400582

[B33] Samuel V. T. Shulman G. I. (2016). The pathogenesis of insulin resistance: integrating signaling pathways and substrate flux. J. Clin. Invest. 126 (1), 12–22. 10.1172/JCI77812 26727229 PMC4701542

[B34] Samuel V. T. Shulman G. I. (2018). Nonalcoholic fatty liver disease as a nexus of metabolic and hepatic diseases. Cell Metab. 27 (1), 22–41. 10.1016/j.cmet.2017.08.002 28867301 PMC5762395

[B35] Samuel V. T. Liu Z. X. Qu X. Elder B. D. Bilz S. Befroy D. (2004). Mechanism of hepatic insulin resistance in nonalcoholic fatty liver disease. J. Biol. Chem. 279 (31), 32345–32353. 10.1074/jbc.M313478200 15166226

[B36] Shimizu K. Nishimuta S. Fukumura Y. Michinaga S. Egusa Y. Hase T. (2022). Liver-specific overexpression of lipoprotein lipase improves glucose metabolism in high-fat diet-fed mice. PLoS One 17 (9), e0274297. 10.1371/journal.pone.0274297 36099304 PMC9469954

[B37] Shimizu K. Ono M. Mikamoto T. Urayama Y. Yoshida S. Hase T. (2024). Overexpression of lysophospholipid acyltransferase, LPLAT10/LPCAT4/LPEAT2, in the mouse liver increases glucose-stimulated insulin secretion. FASEB J. 38 (2), e23425. 10.1096/fj.202301594RR 38226852

[B38] Shindou H. Shimizu T. (2009). Acyl-CoA:lysophospholipid acyltransferases. J. Biol. Chem. 284 (1), 1–5. 10.1074/jbc.R800046200 18718904

[B39] Suganami T. Tanaka M. Ogawa Y. (2012). Adipose tissue inflammation and ectopic lipid accumulation. Endocr. J. 59 (10), 849–857. 10.1507/endocrj.ej12-0271 22878669

[B40] Tanaka Y. Shimanaka Y. Caddeo A. Kubo T. Mao Y. Kubota T. (2021). LPIAT1/MBOAT7 depletion increases triglyceride synthesis fueled by high phosphatidylinositol turnover. Gut 70 (1), 180–193. 10.1136/gutjnl-2020-320646 32253259 PMC7788230

[B41] Teo K. Abeysekera K. W. M. Adams L. Aigner E. Anstee Q. M. Banales J. M. (2021). rs641738C>T near MBOAT7 is associated with liver fat, ALT and fibrosis in NAFLD: a meta-analysis. J. Hepatol. 74 (1), 20–30. 10.1016/j.jhep.2020.08.027 32882372 PMC7755037

[B42] Tian Y. Lu W. Shi R. McGuffee R. Lee R. Ford D. A. (2023a). Targeting phospholipid remodeling pathway improves insulin resistance in diabetic mouse models. FASEB J. 37 (11), e23251. 10.1096/fj.202301122RR 37823674 PMC10575708

[B43] Tian Y. Mehta K. Jellinek M. J. Sun H. Lu W. Shi R. (2023b). Hepatic phospholipid remodeling modulates insulin sensitivity and systemic metabolism. Adv. Sci. (Weinh) 10, e2300416. 10.1002/advs.202300416 37088778 PMC10288282

[B44] Turpin S. M. Nicholls H. T. Willmes D. M. Mourier A. Brodesser S. Wunderlich C. M. (2014). Obesity-induced CerS6-dependent C16:0 ceramide production promotes weight gain and glucose intolerance. Cell Metab. 20 (4), 678–686. 10.1016/j.cmet.2014.08.002 25295788

[B45] Valentine W. J. Yanagida K. Kawana H. Kono N. Noda N. N. Aoki J. (2022). Update and nomenclature proposal for mammalian lysophospholipid acyltransferases, which create membrane phospholipid diversity. J. Biol. Chem. 298 (1), 101470. 10.1016/j.jbc.2021.101470 34890643 PMC8753187

[B46] Valentine W. J. Shimizu T. Shindou H. (2023). Lysophospholipid acyltransferases orchestrate the compositional diversity of phospholipids. Biochimie 215, 24–33. 10.1016/j.biochi.2023.08.012 37611890

[B47] van Meer G. Voelker D. R. Feigenson G. W. (2008). Membrane lipids: where they are and how they behave. Nat. Rev. Mol. Cell Biol. 9 (2), 112–124. 10.1038/nrm2330 18216768 PMC2642958

[B48] Yamaguchi K. Yang L. McCall S. Huang J. Yu X. X. Pandey S. K. (2007). Inhibiting triglyceride synthesis improves hepatic steatosis but exacerbates liver damage and fibrosis in Obese mice with nonalcoholic steatohepatitis. Hepatology 45 (6), 1366–1374. 10.1002/hep.21655 17476695

[B49] Younossi Z. M. Golabi P. Price J. K. Owrangi S. Gundu-Rao N. Satchi R. (2024). The global epidemiology of Nonalcoholic fatty liver disease and nonalcoholic steatohepatitis among patients with type 2 diabetes. Clin. Gastroenterol. Hepatol. 22 (10), 1999–2010 e1998. 10.1016/j.cgh.2024.03.006 38521116

